# Efficient Removal of Nonylphenol Contamination from Water Using Optimized Magnesium Silicate

**DOI:** 10.3390/ma15134445

**Published:** 2022-06-24

**Authors:** Xu Yan, Qicai Zhang, Qinxiong Rao, Shanshan Chen, Xianli Wang, Wei Song, Lin Cheng, Shuhui Guan, Weiguo Song

**Affiliations:** 1 Institute for Agri-Food Standards and Testing Technology, Shanghai Academy of Agricultural Science, Shanghai 201403, China; yanxu024@163.com (X.Y.); qicaizhang@126.com (Q.Z.); qinxiongrao@163.com (Q.R.); cssm100@163.com (S.C.); wangxianli@saas.sh.cn (X.W.); songwei890214@163.com (W.S.); chenglin_8813@126.com (L.C.); 2 College of Food Sciences, Shanghai Ocean University, Shanghai 201306, China; 3 Shanghai Engineer Research Center for Agro-Food Quality and Safety, Shanghai 201403, China

**Keywords:** adsorption, 4−nonylphenol, magnesium silicate, kinetic model, isotherm model

## Abstract

Nonylphenol (NP) is considered to be an environmentally toxic, endocrine-disrupting chemical that affects humans and ecosystems. Adsorption is one of the most promising approaches for the removal of nonylphenol contamination from water. Herein, in order to design an adsorbent with high adsorption capacity, magnesium silicate with different Mg/Si ratios was successfully synthesized by a sol–gel method at 60 °C. Magnesium silicate with a Mg/Si ratio of 1:6 was found to possess the best adsorption performance, with maximum 4−NP sorption 30.84 mg/g under 25 °C and 0.2 g/L adsorbent dose. The adsorption was negatively affected by increasing adsorbent dose and temperature. The kinetics and isotherm of 4−NP adsorption by Mg/Si were well described by the pseudo−second−order and Sips model, respectively, and behavior was proven to be physisorption−enhanced by a chemical effect. Detailed characterization by XRD, BET, and SEM confirmed that the magnesium silicate possesses an amorphous, mesoporous structure. The study will contribute to the applicability of cheap magnesium silicate for removal of NP contamination in water.

## 1. Introduction

Nonylphenol (NP), a toxic xenobiotic compound, is mostly used to produce nonylphenol ethoxylate surfactants, as well as in the manufacturing of antioxidants, pesticide emulsifiers, and resin stabilizers [1,2]. In the case of 4−NP, it tends to accumulate in biological texture resulting from a low solubility in water (5.4 mg/L) and a high hydrophobicity (log Kow 4.48) [3]. It is well−known that NP has simulated estrogen action and potential carcinogenicity [4] by disrupting the interaction of natural hormones with estrogen receptors [5], causing hormonal disorders and influencing reproductive development [6,7]. This pollutant has been listed as one of the priority hazardous substances of the European Union [8]. The semivolatility and mobility of NP resulted in its worldwide detection in surface water, wastewater effluents, and sediments [6,9]. The average concentration in rivers is 4.1 µg/L [10], which represents a threat to humans’ exposure to water. In addition, NP contamination can enter the food chain from water and cause food safety risk [10,11].

Hence, the removal of NP from water has attracted widespread attention and various ways have been adopted to relatively eliminate NP from aquatic systems in recent decades [12,13,14,15], including photocatalytic degradation, membrane technology [16,17], biological methods [1,18,19], and adsorption [15,20,21,22]. The adsorption method is one of the most promising approaches to remove pollutants due to its efficiency, simplicity, and rapid application [21,23]. A large number of adsorbents have been investigated, including activated carbons [24,25], multiwalled carbon nanotubes (MWCNTs) [26], graphene [15,27], and so on. Among these adsorbents, silicate, which is abundant on Earth, has received increasing attention since it is environmentally friendly and has a low cost [28,29]. Since magnesium silicate possesses the advantages of having a high surface area, large pore diameters, and an abundant reserve of functional groups [30], according with most standard adsorbents selection, it has been diffusely used for adsorbing various dyes [31] and heavy metal ions [32] from wastewater. It has been reported that hierarchical nanostructures of magnesium silicate have good adsorption capacities for Pb^2+^, Zn^2+^, and Cu^2+^, with adsorption capacities of 436.68, 78.86, and 52.30 mg/g, respectively [32]. Interestingly, it was found that the ratio of Mg/Si significantly influences the adsorption performance of magnesium silicate [31,33]. The research of synthesized M-S-H samples shows that the Mg/Si ratios of ≤0.5−0.7 are associated with amorphous silica, while Mg/Si ratios of ≥1.0−1.5 are associated with the existence of brucite [33,34,35]. At Mg/Si ratios of 2:1 and by introducing sodium silicate and magnesium salts, it was found that active magnesium silicate could be formed around modified palygorskite (PAL), resulting in a removal capacity of methylene blue (MB) and Cu^2+^ of 527.22 mg/g and 210.64 mg/g, respectively [31]. Magnesium silicate surfaces consist of hydrophobic siloxane groups (≡Si−O−Si≡), hydrophilic groups containing isolated hydroxyl ions (−Mg−OH), individual silanol groups (≡Si−OH), and hydrogen bonds [36,37]. A large increase in the adsorption capacity for chemicals could be achieved by breaking their inert Si−O−Si and Si−O−M bonds and rearranging their crystal structures with a one−pot hydrothermal process [28,29]. By thermal desorption, it was confirmed that NP possibly bonded to oxygen surface groups through hydrogen bonds [38]. As both siloxane and NP are hydrophobic, adsorption can occur through physical processes. Hence, magnesium silicate may be a potential adsorption material for nonylphenol in wastewater. However, the adsorption of 4−NP by magnesium silicate has not yet been reported.

The structure−performance relationship of magnesium silicate has not been clearly elucidated, and its adsorption performance for NP is currently unknown. To address these issues, herein, magnesium silicate was synthesized by the hydrothermal method and the sol−gel method, and the adsorption capacity of 4−NP was compared to find the most efficient magnesium silicate ratio for removal of 4−NP from water.

## 2. Materials and Methods

### 2.1. Materials

Magnesium chloride (MgCl_2_, 99%), guaranteed reagent (GR) grade sodium silicate nonahydrate (Na_2_SiO_3_·9H_2_O), analytical reagent (AR) grade hydrochloric acid (HCl), and sodium hydroxide (NaOH, ≥96%) were purchased from Sinopharm Chemical Reagent Co., Ltd. (Shanghai, China). Additionally, 4−NP (99%) and AR grade nitric acid (HNO_3_) were purchased from Macklin Biochemical Technology Co. (Shanghai, China). Chromatographic−grade methanol was purchased from Merck Company (Darmstadt, Germany). Ultrapure water was obtained from a Milli−Q reagent water system (Millipore, Billerica, MA, USA). The 4−NP stock solutions (5000 mg/g) were prepared by LC−MS grade methanol and then diluting with deionized water. The working standard solution of 4−NP was stored at −20 °C in a brown glass bottle. A small amount of methanol (<1%) was used to avoid cosolvent effects.

The material synthesis was carried out by a constant-temperature magnetic stirrer (SH-HJ4B, Shanghai SiHu Instruments, Shanghai, China). The concentrations of 4−NP were detected by the Waters Acquity UPLC system (Milford, MA, USA) and Xevo triple−quadrupole (Xevo−TQD) AB SCIEX 5500 mass spectrometer (Corp, Framingham, MA, USA). The adsorption was carried out by a shaking incubator (SHJ−2102, Ruskinn, Bridgend, UK). The pH value was adjusted using a pH/Ion Seven Compact (S220, Mettler Toledo, Shanghai, China).

### 2.2. Synthesis of Magnesium Silicate by the Sol−Gel Method

Magnesium silicate was prepared by the dropwise addition of 0.5 mol/L aqueous solution of MgCl_2_ to a 0.5 mol/L aqueous solution of Na_2_SiO_3_·9H_2_O (the volume was controlled according to the Mg/Si ratio of 1:1), stirring continuously in a constant-temperature water bath adjusted to 25 ± 1 and 60 ± 1 °C, respectively. Then, a white suspension was formed by vigorous stirring of both solutions. The pH value of the resulting mixture was then regulated to 9. The previous mixture was stirred successively for 2 h, then condensed and aged overnight at room temperature. The resulting products were then rinsed with deionized water to remove impurities. The dry gels were obtained by placing them in a heating oven for 12 h at 60 °C. The obtained solids were ground and stored at room temperature. Two magnesium silicate samples were individually prepared at different temperatures: sol−gel 25 °C−1Mg:1Si; sol−gel 60 °C−1Mg:1Si. Subsequently, the effect of the Mg/Si ratio was studied following the optimized synthetic way. Additionally, the five samples were prepared on the optimal synthesis route: Mg/Si 3:1, Mg/Si 1:1, Mg/Si 1:3, Mg/Si 1:6, and Mg/Si 1:7.

### 2.3. Synthesis of Magnesium Silicate by the Hydrothermal Method

Another group of magnesium silicate samples was prepared using a hydrothermal method. The same procedure as described above was followed until the hydrothermal treatment step. The value of pH is regulated to 9. The above mixture was transferred to a Teflon−lined autoclave (200 mL), and then maintained in a heating oven at 110 °C for 12 h. Finally, these wet materials were dried at 110 °C for 12 h. The obtained white solids were ground and stored at room temperature. A synthetic magnesium silicate sample was named hydrothermal−1Mg:1Si.

### 2.4. Study on the Adsorption of 4−NP in Aqueous Solution by Magnesium Silicate

#### 2.4.1. Batch Adsorption Experiments for 4−NP in Water

To establish a relationship between the amount of adsorbent with time, the adsorption performance of magnesium silicate for 4−NP was tested by batch techniques under pH 7 and 25 °C. Briefly, a series of 50 mL brown, glass bottles with 30 mL of 4−NP solution (10 mg/L) were put in contact with 30 mg of adsorbents placed in shaking incubator at 200 rpm to achieve adsorption equilibrium. At different intervals, the solution was promptly separated from the adsorbent by a 0.22 µm filter. The above solutions were diluted with methanol and the concentration of residual 4−NP in solution was analyzed by UPLC−MS/MS. All experiments were conducted in parallel three times, each obtained value of experiment was calculated by data from the three parallel, and the experimental values were recorded. The adsorption capacity of 4−NP at equilibrium is expressed and calculated by:

*q_e_* = (*C*_0_ − *C_e_*) *V*/*m* (1)

where *q_e_* (mg/g) is the amount of 4−NP adsorbed per unit mass of adsorbent. *C*_0_ (mg/L) and *C_e_* (mg/L) are the initial and equilibrium concentrations of the 4−NP solution, respectively. *V* (mL) is the volume of the 4−NP solution and *m* (mg) is the mass of the adsorbent. Considering the actual conditions, subsequent experiments were carried out according to the optimal Mg/Si ratio synthesized on the optimal synthesis route.

#### 2.4.2. Comparison with Commercial Materials

The adsorption performance of several materials for 4−NP was surveyed by contacting 30 mL 4−NP solution (10 mg/L) with 30 mg of adsorbents, including magnesium silicate and commercial adsorbents (the details are shown in the supporting information). These series of 50 mL bottles oscillated in a thermostatic vibrator at 25 °C. Then, the remaining concentration of 4−NP at different intervals was measured. The other operation procedures were the same as above.

#### 2.4.3. Effect of Adsorbent Dose on 4−NP Adsorption by Magnesium Silicate

The influence of adsorbent dose (0.1, 0.2, 0.6, 1, 1.6, 2, and 4 g/L) on adsorption capacity was evaluated by sufficiently contacting adsorbents with 10 mg/L 4−NP solution (30 mL). After shaking these series bottles in a thermostatic vibrator at 25 °C for 300 min, the concentration of the residual 4−NP was detected.

#### 2.4.4. Effect of Temperature on 4−NP Adsorption by Magnesium Silicate

The performance of the sample in different temperatures (25, 35, and 45 °C) was carried in 50 mL brown bottles by dispersing adsorbates (0.2 g/L) into 30 mL of 4−NP solution (10 mg/L). The equilibrium concentrations of 4−NP in solution were determined by UPLC−MS/MS. The other operation procedures were the same as above.

#### 2.4.5. Adsorption Kinetics Experiment

To determine the adsorption kinetics, 30 mL of the 4−NP solution (initial concentration of 10 mg/L) was put in contact with 30 mg of five Mg/Si ratio samples at 25 °C and 200 rpm. Then, sampling at intervals (0, 5, 10, 20, 30, 60, 90, 120, 180, 240, and 300 min) to detect the concentrations of the remaining 4−NP in the solution was performed. The other operation procedures were the same as above.

#### 2.4.6. Thermal Adsorption Experiment

A set of 4−NP solutions with 30 mL initial concentrations of 0.1, 1, 3, 5, 7, 9,10, 14, 20, 30, 39, and 51 mg/L (adsorbents dose of 0.2 g/L) was adopted to investigate the adsorption isotherms and thermodynamics for 4−NP. The dose of adsorbents was 0.2 g/L, and the adsorption experiments were carried out at 25, 35, and 45 °C, respectively. Magnesium silicate (dose 0.2 mg/L) was added into 50 mL brown bottles filled with 30 mL of 4−NP solution. These brown bottles were oscillated in a thermostatic shaker for 300 min at 25, 35, and 45 °C. The other operation procedures were the same as above.

#### 2.4.7. Recyclability of Magnesium Silicate

The recyclability was studied in 50 mL brown glass bottles with 10 mg/L 4−NP solution (30 mL) by dispersing optimal adsorbents (1.6 mg/L). These brown bottles were shaken at 25 °C (200 rpm) for 300 min. The adsorbent, Mg/Si 1:6, was then separated by centrifugation (3900 rpm, 5 min). The 4−NP−containing adsorbents were eluted several times by acetonitrile until the 4−NP could not be detected from the eluant by HPLC−MS/MS. Finally, the adsorbents, without 4−NP, were collected and dried at 60 °C for 12 h. The obtained adsorbent was reused to conduct the following adsorption experiments.

### 2.5. Characterization

The details and instruments are shown in the supporting information.

## 3. Results and Discussion

### 3.1. Synthesis Route and Characterization of Magnesium Silicate

#### 3.1.1. Comparison of Synthesis Route

**Figure 1:**
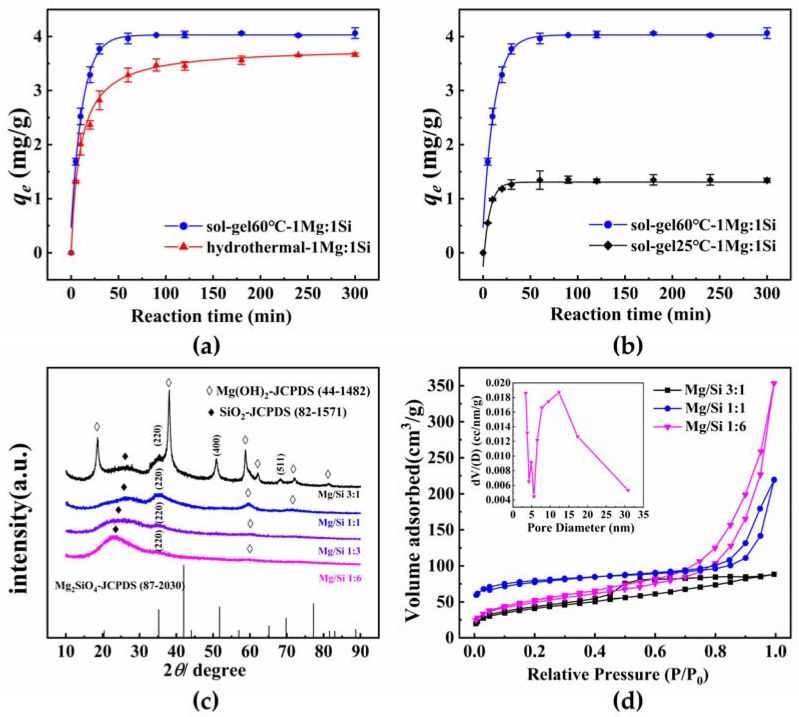
Influence of synthesis methods (**a**) and reaction temperature of sol−gel method (**b**) for synthesis magnesium silicate on adsorption capacity for 4−NP. XRD pattern (**c**) and N_2_ adsorption−desorption (**d**) with different Mg/Si ratios.

Synthesis of magnesium silicate was performed using sol−gel and hydrolysis. In Figure 1a, it shows that the adsorption amount of 4−NP rapidly increased with prolonging the contact time for adsorbents synthesized by sol−gel and hydrothermal methods. Figure 1b depicts the adsorption amounts of 4−NP for adsorbents synthesized by the sol−gel method at 25 and 60 °C, respectively. The sample synthesized by the sol−gel method reaches adsorption equilibrium faster than that prepared by the hydrothermal method. The adsorbent of sol−gel 60 °C−1Mg:1Si has a slightly higher equilibrium amount (*q_e_*) compared to hydrothermal−1Mg:1Si. In Figure 1b, the adsorption capacity increases from 1.34 to 4.06 mg/g as the reaction temperature from the sol−gel method increases from 25 to 60 °C. It is concluded that the synthesis temperature plays a key role in the fabrication of efficient magnesium silicate by the sol−gel method. Therefore, the subsequent research was carried out with magnesium silicate synthesized by the sol−gel method at 60 °C.

#### 3.1.2. Comparison of Synthesis Route

XRD: Herein, the structure, morphology, and pore characteristics of magnesium silicate were investigated using XRD, BET, and SEM. Figure 1c presents the XRD patterns of four magnesium silicates with different Mg/Si ratios. The samples show successions of typical peaks of Mg_2_SiO_4_, well−matched with JCPDS card no. 87-2030, at 2θ~35.540°, 51.140°, and 68.206° for (220), (400), and (511), respectively. The intensity of characteristic peaks, however, is slightly less than that reported according to Temuujin et al. [39]. Notably, these magnesium silicate features become poorer with an increasing silicon fraction, suggesting that the materials have an amorphous nature. A broad peak appears at 2θ~25° due to the generation of SiO_2_, which corresponds to the report by El-Naggar et al. [40]. It is indicated that the Mg/Si ratio has a vital influence on the crystallite structure of the magnesium silicate sample.

BET: Figure 1 showed the N_2_ adsorption–desorption isotherm, obtained at 77 K, of magnesium silicate with three Mg/Si ratios at 77 K, and the pore size distribution of Mg/Si 1:6. Based on classification of IUPAC, it can be seen that Mg/Si 1:6 exhibits an obvious type H3 hysteresis loop generated by the loosely coherent aggregates of a plate−like substance [31], which agree with the SEM image observed in Figure S2. Furthermore, the Mg/Si 3:1 is noted as type H1 hysteresis loop, which is typically given by the uniform mesopores with narrow distribution range [41]. Mg/Si 1:1 exhibits a hysteresis loop of type H4, somewhat similarly to type H3, while the more significant increase in adsorption at low P/P_0_ is related to the existence of micropores [41]. It can be noted that the increase in Si proportion may be unfavorable to the constitution of uniform mesopores. Since any adsorption equilibrium of N_2_ is observed at high P/P_0_ values, the pseudo−type II isotherm caused by delayed capillary condensation can describe the isotherm of magnesium silicate [42]. As the P/P_0_ value reaches 0.8, a sharp rise was noticed in the adsorption amount due to multilayer adsorption of N_2_ on the mesopores [43]; the mean pore diameter is shown in Table 1.

The surface area of the Mg/Si 1:6 (117.7 m^2^/g) is relatively higher than that of the 1:1 (41.90 m^2^/g) and 3:1 (98.58 m^2^/g). On the other hand, the average pore diameter for the Mg/Si 1:6 (12.30 nm) is also larger than the Mg/Si 1:1 (1.706 nm) and Mg/Si 3:1 (3.827 nm). The observed increase in the pore volume can be attributed to the formation of loosely coherent aggregates with a decrease in Mg^2+^ content.

SEM: Figure S2 exhibits representative SEM images of the magnesium silicate fabricated with different Mg/Si ratios. Some plate−shaped or amorphous matter could be observed. It is obvious that the fabricated samples show a high aggregation tendency upon drying. It is confirmed that a nonuniform sample was obtained. The morphology significantly varied with increasing Si ions from a disordered, plate-like structure for Mg/Si 3:1 to loosely coherent aggregates for Mg/Si 1:6. The space between these aggregates results in the formation of larger pores, i.e., a high proportion of silicon favors a high surface area.

### 3.2. Optimization of Mg/Si Ratio

**Figure 2:**
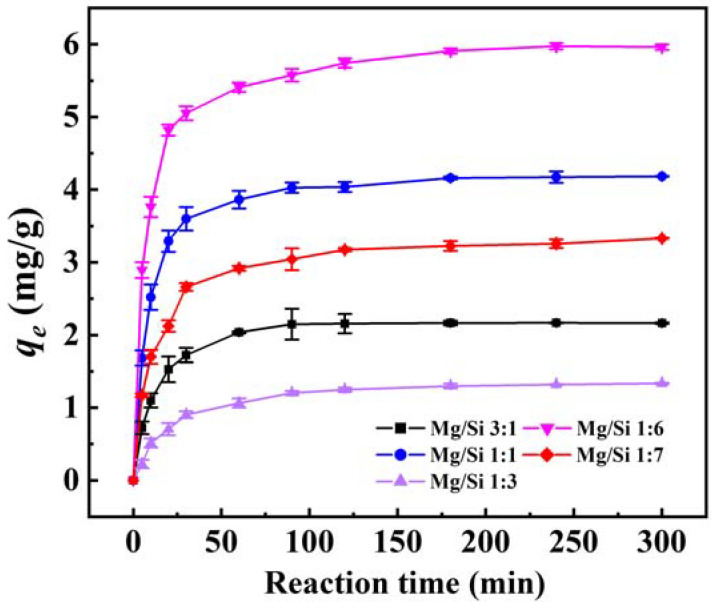
Influence of Mg/Si ratio on adsorption capacities of magnesium silicate for 4−NP.

Figure 2 shows the change of adsorption amounts of adsorbents with Mg/Si ratios of 3:1, 1:1, 1:3, 1:6, and 1:7 for 4−NP versus contact time. With the extension of contact time, the adsorption amounts of the 4−NP was increased rapidly. The adsorption equilibrium can be virtually completed within the first half hour, due to the large solid−liquid concentrate gradient and abundant active sites at the beginning of contact. The adsorbent with the Mg/Si ratio of 1:6 shows a higher adsorption capacity (5.97 mg/g) for 4−NP than the other Mg/Si ratios. The adsorption amounts when reaching the adsorption equilibrium were 4.06, 3.32, 2.17, and 1.37 mg/g for Mg/Si ratios of 1:1, 1:7, 3:1, and 1:3, respectively. In addition, the equilibrium adsorption capacities of Mg/Si 1:6 were higher than that of other commercial materials at the same condition (Figure S3). Additionally, the cost of Mg/Si 1:6 was 1500 times higher than that of g−C_3_N_4_ and basically equal to the commercial attapulgite.

As shown in Figure 1c, when a smaller amount of Mg^2+^ is used, the amorphous magnesium silicate particles can grow easily. It is inferred that the amorphous nature of Mg/Si 1:6 is beneficial in improving the adsorption performance. These results suggest that the adsorption equilibrium amount has a strong relationship with the Mg/Si ratio. The Mg/Si 1:6 also shows the fastest adsorption rate for 4−NP. The adsorptive property of the Mg/Si 1:6 is the best one due to the related surface area and highest pore size. As a result, the material pore diameter and surface area are important for the efficient removal of 4−NP from water. Hence, it is necessary to develop adsorbents with high specific surface area and large pore diameter by controlling the synthesis methods to enhance adsorption capacity.

### 3.3. Effect of Dosage and Temperature

**Figure 3:**
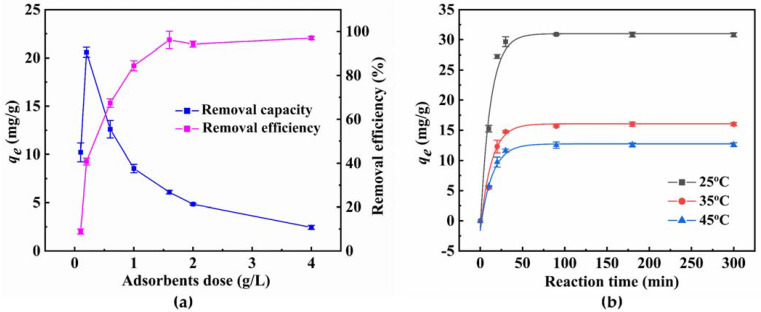
Influence of adsorbent dose (**a**) and temperature (**b**) on 4−NP uptake for Mg/Si 1:6.

Adsorbent dose: The change in 4−NP uptake with adsorbent dose is shown in Figure 3a. With an elevated dosage of Mg/Si 1:6 powder, the removal efficiency of 4−NP increased significantly. The removal percentage of 4−NP is almost close to 100% with the adsorbent dose ≥ 1.6 g/L. In addition, an increase in adsorption amounts of 4−NP from 10.20 to 20.59 mg/g was observed when doubling the adsorbent dose to 0.2 g/L. This increase in the uptake of 4−NP is probably due to an increase in active sites. However, the adsorption amount of 4−NP decreased sharply until the dosage was 1.6 g/L. Subsequently, the adsorption amount of 4−NP proceeded to decline at a slower rate with increasing magnesium silicate dose. Since the initial concentration is the dynamic of solid−liquid mass transfer, the adsorption capacity of 4−NP decreased with the decrease in concentration gradient between the liquid and solid [44]. Additionally, this may also be due to the reduction of surface area caused by the agglomeration of adsorbent particles [44]. The result showed that the adsorption of 4−NP by the magnesium silicate depends on the dose, and a suitable dose is 0.2 g/L.

Adsorption temperature: Temperature is also a significant factor that influences adsorption. The effect of temperature on the removal of 4−NP is presented in Figure 3b. It can be seen that 4−NP adsorption amount gradually grows as temperature decreases. The adsorbent showed an excellent adsorption capacity for 4−NP at 25 °C and the maximum adsorption amount was 30.84 mg/g. As a result, the adsorption of 4−NP by magnesium silicate was related to temperature and has an exothermic nature [45]. Therefore, the adsorption of 4−NP was favored at a relatively low temperature of 25 °C. It is proven that magnesium silicate can meet the requirement of removing 4−NP in wastewater at a normal temperature.

### 3.4. Adsorption Mechanism of 4−NP by Magnesium Silicate

**Figure 4:**
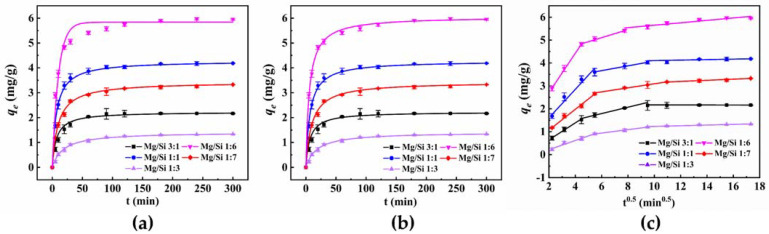
Removal of 4−NP by Mg/Si 1:6 fitted with nonlinear pseudo−first−order kinetic model (**a**), nonlinear pseudo−second−order kinetic model (**b**), and intraparticle diffusion model (**c**).

Kinetics: To examine the corresponding adsorption processes of 4−NP on five samples, pseudo−first−order, pseudo−second−order, and intraparticle diffusion were also adopted to describe the adsorption behavior. The nonlinear fitting of the above models for 4−NP is shown in Figure 4.

The adsorption rate and efficiency were determined from adsorption kinetics. In Table 2, the sample of Mg/Si 1:6 shows the relatively fast adsorption rate for 4−NP, resulting from the larger pore size. Considering the correlation coefficients (*R*^2^), the *R*^2^ (0.9770−0.9914) of pseudo−second−order kinetic model for adsorption 4−NP were slightly higher than that of the pseudo−first−order kinetic model (0.9957–0.9980). Additionally, the calculated adsorption amounts of 4−NP at equilibrium were close to that of the experimental data, and the maximum value of theoretical *q_e_* was 6.034 mg/g at Mg/Si 1:6. The pseudo−second−order kinetic could better fit the adsorption process.

Furthermore, Figure 4c depicts the behavior that the 4−NP adsorption on five samples fits with the intraparticle diffusion (IPD) model. Here, the IPD model could also fit well with the adsorption process of 4−NP by magnesium silicate, divided into three stages, which confirmed that IPD played a significant role in 4−NP adsorption. Among the five samples, the Mg/Si 1:6 showed the largest intercept value due to its highest surface area [32]. Additionally, it was observed that both Mg/Si 1:1 and Mg/Si 1:6 had a shorter intraparticle diffusion process due to the larger pore size [32]. The pore structure parameters affecting the IPD process of 4−NP on magnesium silicate mainly include specific surface area and average pore size.

**Figure 5:**
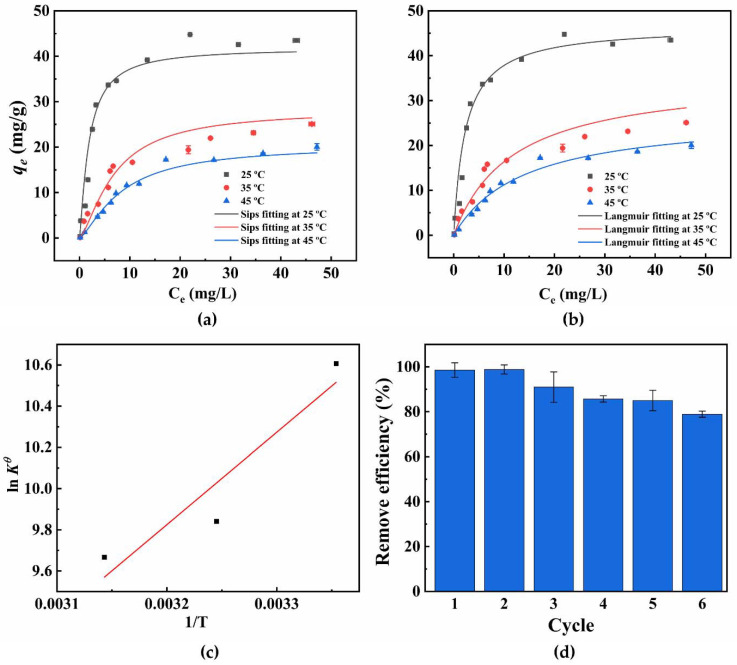
4−NP removal by Mg/Si 1:6 with Sips isothermal fitting (**a**). Langmuir isothermal fitting (**b**). Plot of ln *K^θ^* versus 1/T (**c**). Recycling of Mg/Si 1:6 in the adsorption of 4−NP (**d**).

Isotherm: Here, the four widely used adsorption isotherm models, Langmuir, Freundlich, Sips, and Temkin, are used to clarify the essence of the adsorption process for 4−NP under different temperatures (25, 35, and 45 °C). The nonlinear fitting graphs of the Sips and Langmuir isotherm models are shown in Figure 5a,b. The parameters of those models are presented in Table 3. It was observed that the process of equilibrium adsorption could be well modeled by Sips (*R*^2^, 0.9423−0.9924) and Langmuir (*R*^2^, 0.9413−0.9896) isotherms, and the adaptation sequence of the four isothermal models was as follows: Sips, Langmuir, Freundlich, and Temkin isotherm. Notably, *R*^2^ was the adjusted determination coefficient, which could inhibit the multiparameter isotherms [46]. The Sips model originates from the combination of the Langmuir and Freundlich isotherms, and it is considered that adsorption occurs on heterogeneous surfaces. Moreover, it is predicted that the monolayer has adsorption characteristics at higher adsorbate concentrations, that is, there is a finite limit.

As shown in Table 3, the Freundlich model (*R*^2^, 0.8900−0.9635) showed a better simulation of 4−NP adsorption performance than the Temkin model (*R*^2^, 0.7932−0.8600). The values of 1/n at different temperatures lies in the range of 0.1−1, indicating adsorption was easy [47]. Furthermore, the maximum adsorption capacity *q_m_* of 4−NP calculated from the Langmuir model at 25, 35, and 45 °C was 46.63, 35.61, and 26.89 mg/g, respectively, which was higher than that of 4−NP on some adsorbents (e.g., activated carbon, 39.49 mg/g) [48]. As shown in Table 3, it was observed that *R*^2^ value of Temkin isotherm was lower than 0.9070, which suggested that there was a weak interior molecular force between Mg/Si 1:6 and 4−NP in adsorption processes [23]. Therefore, the adsorption of 4−NP by Mg/Si 1:6 that occurred on heterogeneous surfaces was easy. Additionally, it was a monolayer adsorption without other interactions (e.g., intermolecular interactions between adsorbate) at a high concentration state.

Thermodynamics: Adsorption mechanism can be inferred from thermodynamics. Firstly, the Sips equilibrium constant (*α_s_*) was transformed to be dimensionless [49]. The relationship between ln *K^θ^* and 1/T was exhibited in Figure 5c. Table 4 lists the calculated values of thermodynamic parameters in standard state. Standard state thermodynamic parameters were calculated using:

ln *K^θ^* = Δ*S^θ^*/R − Δ*H^θ^*/RT (2)

*K^θ^* = *α_s_* × molecular weight of 4−NP × 1000 (3)

Δ*G^θ^* = Δ*H^θ^* − TΔ*S^θ^* (4)

where α_s_ is the standard state Sips isotherm constant, R is the universal gas constant (8.314 J/mol·K), T is the absolute temperature (K), ΔG^θ^ is the Gibbs free energy change (kJ/mol), ΔS^θ^ is entropy change (kJ/K·mol), and ΔH^θ^ is enthalpy change (kJ/mol). With the increase in temperature, the ΔG^θ^ values decreased gradually, which indicates that the adsorption became less feasible [45]. The exothermic nature of the adsorption process can be confirmed by the negative ΔH^θ^. Besides, physisorption was involved in the adsorption process when ΔH^θ^ had a value less than 40 kJ/mol. The value of Gibbs free energy that lied in −47.83~−48.55 kJ/mol demonstrated that the adsorption was physisorption enhanced by a chemical effect. The negative value of ΔS^θ^ presented that magnesium silicate had an affinity for 4−NP molecules in aqueous solution, which makes 4−NP molecules more regular.

Recyclability of Mg/Si 1:6: In addition, studying the reusability of materials in specific applications was one of the most important parameters to evaluate adsorbents. The recoverability of Mg/Si 1:6 after six adsorption−desorption iterations is shown in Figure 5d. The decline in removal efficiency (18%) indicated that the Mg/Si 1:6 possessed good reusability and stability. As a promising adsorbent, the magnesium silicate could be used to remove 4−NP from wastewater.

## 4. Conclusions

In summary, it was found that optimized magnesium silicate, Mg/Si 1:6, was an efficient adsorbent to remove 4−NP from water, with its highest adsorption capacity reaching 30.84 mg/g. Through characterization, the optimized adsorbent possessed the structure of amorphous and mesoporous with a higher surface area (117.7 m^2^/g). Adsorption was negatively affected by increasing temperature and adsorbent dose. The adsorption behavior conformed to the pseudo−second−order kinetic model, while the calculated adsorption amounts of 4−NP in equilibrium were close to the experimental value. The adsorption isotherm accords with the Sips isotherm model. The thermodynamic studies confirmed that the adsorption mechanism was physisorption under the effect of chemical enhancement. Moreover, the adsorbent came from environmentally compatible elements, such as Si and Mg, and was a new adsorbent with low cost, high efficiency, nontoxicity, and environmental protection. Therefore, magnesium silicate exhibited a good adsorption performance for 4−NP, indicating that magnesium silicate is a promising material to remove NP from wastewater.

## Figures and Tables

**Table 1:** BJH analysis of the magnesium silicate with different ratios of Mg/Si.

Sample	Surface Area (m^2^/g)	Pore Volume (cm^3^/g)	Average Pore Diameter (nm)
Mg/Si 3:1	98.58	0.1030	3.827
Mg/Si 1:1	41.90	0.2310	1.706
Mg/Si 1:6	117.7	0.5080	12.30

**Table 2:** Adsorption kinetic parameters of 4−NP.

Samples	Pseudo−First−Order				Pseudo−Second−Order		
	*q_e_*_,*exp*_ (mg/g)	*q_e_*_,*cal*_ (mg/g)	*K*_1_ (min^−1^)	*R* ^2^	*q_e_*_,cal_ (mg/g)	*K*_2_ (g/mg·s)	*R* ^2^
Mg/Si 3:1	2.075	2.137	0.06618	0.9914	2.313	0.04161	0.9957
Mg/Si 1:1	4.063	4.044	0.09349	0.9895	4.323	0.03283	0.9978
Mg/Si 1:3	1.374	1.278	0.04023	0.9869	1.438	0.03526	0.9959
Mg/Si 1:6	5.961	5.676	0.1129	0.9745	6.034	0.02930	0.9980
Mg/Si 1:7	3.330	3.154	0.06804	0.9770	3.417	0.02876	0.9964

**Table 3:** Adsorption isotherm parameters for the adsorption of 4−NP.

	Parameter	Temperature		
		25 °C	35 °C	45 °C
Sips Model	*K_s_* (L/g)	19.23	1.782	1.111
	*α_s_* (L/mg)	0.4600	0.0632	0.05431
	*β_s_*	1.239	1.430	1.376
	*R* ^2^	0.9891	0.9423	0.9924
Langmuir Model	*K_L_* (L/mg)	0.4297	0.08525	0.07162
	*q_m_* (mg/g)	46.63	35.61	26.89
	*R* ^2^	0.9879	0.9413	0.9896
Freundlich Model	*K_F_* (L/g)	13.84	3.8593	2.590
	1/n	0.4625	0.5875	0.6146
	*R* ^2^	0.9218	0.8900	0.9635
Temkin Model	*K_T_* (L/g)	7.482	3.314	2.366
	*A* (J/mol)	8.010	4.634	3.796
	*R* ^2^	0.9070	0.9154	0.8327

**Table 4:** Values of Δ*G^θ^*, Δ*H^θ^*, and Δ*S^θ^*.

T (K)	*K^θ^*	Δ*G^θ^* (kJ/mol)	Δ*H^θ^* (kJ/mol)	Δ*S^θ^* (kJ/K·mol)
25 °C	101361	−47.83	−37.06	−0.03611
35 °C	13926.1	−48.187		
45 °C	11967.2	−48.55		

## Data Availability

Not applicable.

## Supplementary Materials

The following supporting information can be downloaded at: https://www.mdpi.com/article/10.3390/ma15134445/s1, Table S1: MS/MS parameters for 4−NP, Figure S1: LC-MS/MS chromatogram of 4−NP, Figure S2: The SEM images of magnesium silicate with different ratio of Mg/Si, Figure S3: Adsorption of 4−NP by Mg/Si 1:6 versus other commercial materials.
