# Supplementary material for: Efficient Removal of Nonylphenol Contamination from Water Using Optimized Magnesium Silicate
Source: Materials (Basel). 2022 Jun 24;15(13):4445. doi: 10.3390/ma15134445 (PMC9267514; doi:10.3390/ma15134445)
Supplement: Supplementary file 1 [file materials-15-04445-s001.zip › materials-1754000-supplementary.pdf]

Article

Efficient Removal of Nonylphenol Contamination from Water Using Optimized Magnesium Silicate

Xu Yan [1,2], Qicai Zhang [1,3], Qinxiong Rao [1,3], Shanshan Chen [1,3], Xianli Wang [1,3], Wei Song [1,3], Lin Cheng [1,3],
Shuhui Guan [1,3,*] and Weiguo Song [1,3,*]

[1] Institute for Agri−Food Standards and Testing Technology, Shanghai Academy of Agricultural Science,
Shanghai 201106, China; yanxu024@163.com (X.Y.); qicaizhang@126.com (Q.Z.);
qinxiongrao@163.com (Q.R.); cssm100@163.com (S.C.); wangxianli@saas.sh.cn (X.W.);
songwei890214@163.com (W.S.); chenglin_8813@126.com (L.C.)

[2] College of Food Sciences, Shanghai Ocean University, Shanghai 201306, China

[3] Shanghai Engineer Research Center for Agro−Food Quality and Safety, Shanghai 201403, China

* Correspondence: shuhuiguan@163.com (S.G.); songweiguo@saas.sh.cn (W.S.); Tel.: +86−21−62202796 (W.S.)

Detection of 4-nonylphenol

The concentrations of 4-NP were analyzed using a Waters Acquity UPLC system (Milford, MA, USA) connected to a Xevo triple-quadrupole (Xevo-TQD) AB SCIEX 5500 mass spectrometer (Corp Framingham, MA, USA) equipped with an electrospray ionization source (ESI). The Waters Acquity UPLC BEH C18 column (50 mm×2.1 mm, 1.7 mm particle size) (Milford, MA, USA) was used for LC separation. Ammonia (0.1%) and methanol were used as mobile phases A and B, respectively. The solvent programming was set to 0.0–2.0 min with 30% A, 2.0–5.5 min with 95% A and 5.5–6.0 min with 30% A. The flow rate, column temperature and injection volume were set at 0.35 mL/min, 40 °C and 2 μL, respectively. The MS/MS detection was carried out in negative mode (ESI-) for 4-NP. The parameter status of the ESI was as follows: curtain gas: 35 psi; ion spray voltage: 5500 V; temperature: 500 °C, ion source gas 1: 50 psi; ion source

gas 2: 50 psi; collision gas: "medium". The parameter status of the MS featured an entrance potential of 10 V and a collision cell exit potential of 9 V. The calibration curves for the analytes were obtained by a linear regression analysis on standard solutions from 1 to 100 μg /L. The parent ion of 4-NP was set at m/z = 219.3, corresponding to the major product ions at m/z = 133.0. The retention time of 4-NP was 2.71 min (Figure S1). Quantitative analysis was performed in the multi-reaction monitoring mode and the optimal MS/MS parameters of 4-NP are shown in Table S1.

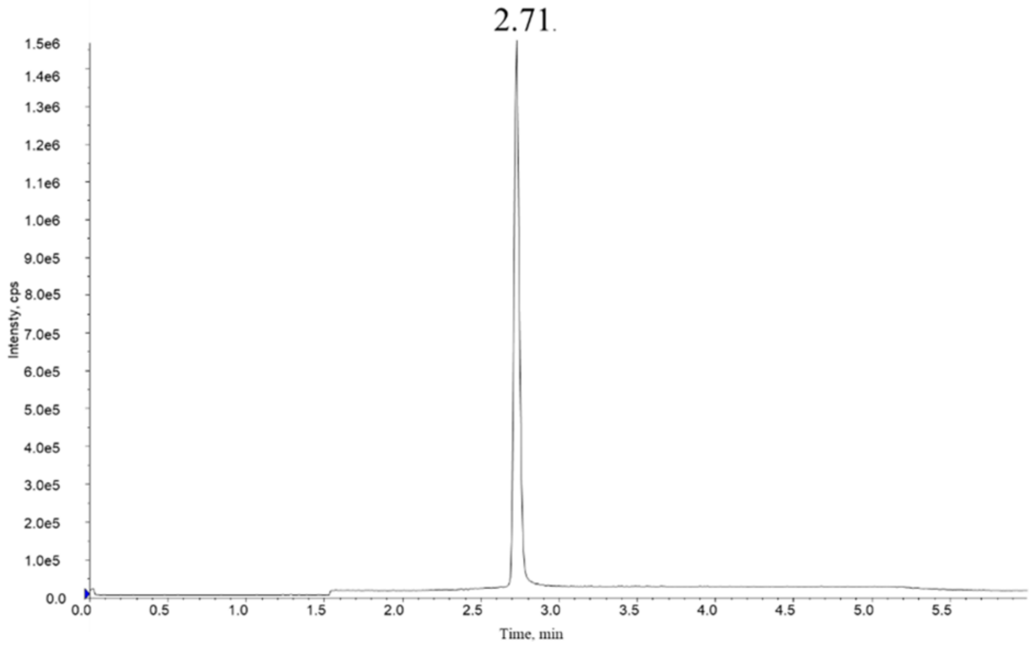

Figure S1. LC-MS/MS chromatogram of 4-NP.

Table S1. MS/MS parameters for 4-NP.

**Analyte**	**Molecular Formula**	**MW**	**t_R (Min)**	**Ion Source**	**Precursor (m/z)**	**Product (m/z)**	**DP(V)**	**CE(eV)**
4-NP	$C_{15}H_{24}O$	220.35	2.71	ESI-	219.3	133.0*,147.0	-89.0	-38.0

Note: MW- molecular weight; T_R- retention time; DP- Declustering potential ; CE-Collision energy. *Quantification ion

Characterization

The crystal structure and composition of the samples were determined by X-ray diffraction (XRD, Bruker D8 Advance, Germany). N2 adsorption-desorption isotherms were constructed at 77K using a micropore physisorption analyzer (Micromeritics ASAP2460, American). The specific surface area, pore volume and pore size distribution data of samples were calculated based on the Barrett-Joyner-Halenda (BJH) method. The morphological changes of magnesium silicate were observed and analyzed by scanning electron microscope (SEM, Hitachi Regulus8100, Japan).

SEM image

The SEM image of the magnesium silicate with the Mg/Si ratio 3:1, 1:1 and 1:6 is shown in Figure S2.

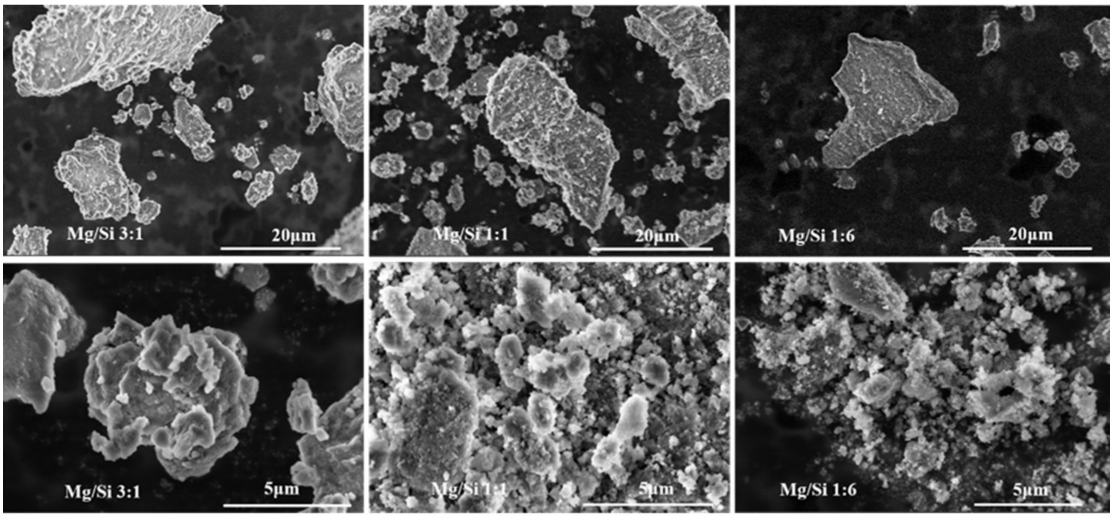

Figure S2. The SEM images of magnesium silicate with different ratio of Mg/Si.

Comparison with commercial materials

Commercial materials: Active carbon (AC, 99%) and carboxylic multi-walled carbon nanotubes (CMWNTs, 10–30 μm, >98%) were obtained from Aladdin Reagent Inc. (Shanghai, China). Graphitic carbon nitride (g-C_3N_4, 99%) was purchased from XFNANO Materials Tech Co. Ltd. (Nanjing, China). Sepiolite (400 mesh) and PAL (200 mesh) were purchased from Beijing Runzekang_Bioscience Co., Ltd. (Beijing, China).

From Figure 7, the adsorption equilibria of the six materials were reached within 120 min. Among them, the adsorption equilibrium of Mg/Si 1:6, CMWNTs and PAL was first reached at 30 min. The maximum time for the AC to reach adsorption equilibrium was 120 min. The Mg/Si 1:6 adsorption effect is the best, with an adsorption amount of 6.38 mg/g. g-C_3N_4 had the second best removal rate of 5.97 mg/g and the third best was the CMWNTs with 4.27 mg/g. Compared with the other materials, the adsorption capacity of Mg/Si 1:6 for 4-NP was 6.38 mg/g, which was significantly higher than that of AC (3.14 mg/g), sepiolite (2.89 mg/g) and PAL (1.87 mg/g). Additionally, the adsorption capacity of Mg/Si 1:6 was two times that of AC. Therefore, the synthesized magnesium silicate has good adsorption properties.

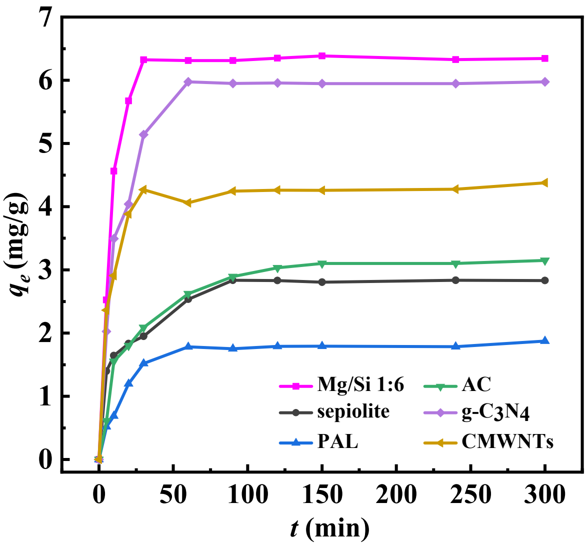

Figure S3. Adsorption of 4-NP by Mg/Si 1:6 versus other commercial materials.
